# Glucagon-like peptide-1 receptor activation in the ventral tegmental area attenuates cocaine seeking in rats

**DOI:** 10.1038/s41386-018-0010-3

**Published:** 2018-02-14

**Authors:** Nicole S. Hernandez, Kelsey Y. Ige, Elizabeth G. Mietlicki-Baase, Gian Carlo Molina-Castro, Christopher A. Turner, Matthew R. Hayes, Heath D. Schmidt

**Affiliations:** 1 Neuroscience Graduate Group, Perelman School of Medicine, University of Pennsylvania, Philadelphia, PA 19104 USA; 2 Department of Psychiatry, Perelman School of Medicine, University of Pennsylvania, Philadelphia, PA 19104 USA; 3 Department of Biobehavioral Health Sciences, School of Nursing, University of Pennsylvania, Philadelphia, PA 19104 USA

## Abstract

Novel molecular targets are needed to develop new medications for the treatment of cocaine addiction. Here we investigated a role for glucagon-like peptide-1 (GLP-1) receptors in the reinstatement of cocaine-seeking behavior, an animal model of relapse. We showed that peripheral administration of the GLP-1 receptor agonist exendin-4 dose dependently reduced cocaine seeking in rats at doses that did not affect ad libitum food intake, meal patterns or body weight. We also demonstrated that systemic exendin-4 penetrated the brain where it putatively bound receptors on both neurons and astrocytes in the ventral tegmental area (VTA). The effects of systemic exendin-4 on cocaine reinstatement were attenuated in rats pretreated with intra-VTA infusions of the GLP-1 receptor antagonist exendin-(9–39), indicating that the suppressive effects of systemic exendin-4 on cocaine seeking were due, in part, to activation of GLP-1 receptors in the VTA. Consistent with these effects, infusions of exendin-4 directly into the VTA reduced cocaine seeking. Finally, extinction following cocaine self-administration was associated with decreased preproglucagon mRNA expression in the caudal brainstem. Thus, our study demonstrated a novel role for GLP-1 receptors in the reinstatement of cocaine-seeking behavior and identified behaviorally relevant doses of a GLP-1 receptor agonist that selectively reduced cocaine seeking and did not produce adverse effects.

## Introduction

One hallmark of cocaine addiction is the high rate of relapse to compulsive drug use during periods of abstinence [1]. Indeed, the most difficult aspect of treating cocaine addiction is preventing relapse [2]. Unfortunately, there are currently no effective FDA-approved treatments for cocaine relapse, which continues to be a significant public health concern. Thus, there is a clear need to identify and develop novel pharmacotherapies for cocaine addiction.

Glucagon-like peptide-1 (GLP-1) is an incretin hormone and neuropeptide that is produced peripherally by intestinal L cells and centrally by preproglucagon (PPG) neurons within the nucleus tractus solitarius (NTS) of the caudal brainstem [3, 4]. GLP-1 receptor ligands are FDA-approved for treating type II diabetes mellitus and obesity based on their ability to increase insulin production and reduce food intake [5–8]. In addition, a growing literature indicates that peripheral administration of GLP-1 receptor agonists attenuates drug-associated behavioral responses including cocaine-induced conditioned place preference (CPP) and the locomotor-activating effects of cocaine [9–11]. However, no studies to date have examined the efficacy of GLP-1 receptor agonists to reduce the reinstatement of cocaine-seeking behavior, an animal model of relapse [12, 13].

GLP-1 receptors are expressed throughout the brain including the ventral tegmental area (VTA), a nucleus known to play a critical role in cocaine-seeking behavior [14–16]. Our lab recently identified a novel role for central GLP-1 receptors in cocaine self-administration in rats [17]. Specifically, we found that activation of GLP-1 receptors in the VTA attenuates cocaine self-administration [17]. While these results suggest that GLP-1 receptors may represent a novel target for cocaine addiction pharmacotherapies, the role of central GLP-1 signaling in cocaine seeking is unknown.

The present study had four main goals: (1) to assess the ability of systemic injections of the GLP-1 receptor agonist exendin-4 to attenuate the reinstatement of drug-seeking behavior elicited by an acute priming injection of cocaine or re-exposure to conditioned cues previously associated with cocaine self-administration; (2) to determine if the effects of systemic exendin-4 on cocaine seeking are due, in part, to activation of GLP-1 receptors in the VTA; (3) to investigate the effects of direct activation of VTA GLP-1 receptors on cocaine seeking; and (4) to characterize the effects of cocaine self-administration and subsequent extinction on GLP-1 receptor mRNA expression in the VTA and PPG mRNA expression in the NTS. Our findings support the hypothesis that GLP-1 receptor activation is sufficient to attenuate the reinstatement of cocaine-seeking behavior. Moreover, we identified doses of the GLP-1 receptor agonist exendin-4 that significantly attenuated cocaine seeking and did not produce adverse malaise-like effects or reduce feeding behaviors. These results indicate a novel role for central GLP-1 receptors in cocaine-seeking behavior and suggest that pharmacotherapies targeting GLP-1 receptors may represent novel approaches for treating cocaine relapse.

## Materials and Methods

Details regarding all drugs used, surgeries, cocaine and sucrose self-administration/extinction/ reinstatement, quantitative PCR and immunohistochemical analyses are available in the Supplement.

### Animals and housing

Male Sprague-Dawley rats (*Rattus norvegicus*) weighing 225–250 g were obtained from Taconic Laboratories. Rats were individually housed with food and water available ad libitum in their home cages. A 12/12 h light/dark cycle was used with the lights on at 7:00 am. All experimental procedures were performed during the light cycle. The experimental protocols were consistent with the guidelines issued by the US National Institutes of Health and were approved by the University of Pennsylvania’s Institutional Animal Care and Use Committee.

### Cocaine reinstatement

To determine whether systemic administration of a GLP-1 receptor agonist reduces cocaine seeking and penetrates the brain, initial studies utilized the GLP-1 receptor agonist exendin-4 tagged with fluorescein (fluoro-Ex-4). In addition to binding GLP-1 receptors in vitro and in vivo, fluoro-Ex-4 produces behavioral responses identical to unlabeled exendin-4 [18, 19]. Once cocaine-taking behavior was extinguished, rats were pretreated with vehicle or 3.0 µg/kg fluoro-Ex-4 (i.p.) 1 h prior to an acute priming injection of cocaine (10 mg/kg, i.p.). Rats were then placed immediately into the operant conditioning chambers and a two-hour reinstatement test session commenced. Separate groups of rats were used in the fluoro-Ex-4 dose–response study to identify doses that attenuate cocaine reinstatement, and are not associated with adverse malaise-like effects [20, 21]. In this experiment, rats were pretreated with vehicle, 0.01, 0.1, and 0.2 µg/kg fluoro-Ex-4 (i.p.) 1 h prior to a 10 mg/kg priming injection of cocaine and subsequent reinstatement test sessions. Using a within-subjects design, each rat served as its own control and fluoro-Ex-4 doses were counterbalanced to avoid rank order effects of drug treatment. 24 h after each treatment, rats were weighed to confirm that 0.01, 0.1, and 0.2 µg/kg doses of fluoro-Ex-4 do not affect body weight.

To determine if the effects of peripherally administered exendin-4 on cocaine seeking are due, in part, to activation of GLP-1 receptors in the brain, the GLP-1 receptor antagonist exendin-(9–39) was microinjected into the VTA prior to systemic administration of fluoro-Ex-4. Obturators were removed from the guide cannulae and 33 gauge stainless steel microinjectors (18 mm; 2 mm projection, Plastics One) were inserted. Using a within-subjects design, rats were infused bilaterally with vehicle or 10 µg/100 nl exendin-(9–39) directly into the VTA. Microinjectors were left in place for an additional one minute following infusions in order to allow for diffusion of the drug solution away from the tips of the microinjectors. Rats were then placed back in their home cages. 30 min later rats received a systemic injection of vehicle or 3.0 µg/kg fluoro-Ex-4 (i.p.) and returned back to their home cages. 1 h later rats received an acute 10 mg/kg priming injection of cocaine prior to a reinstatement test session.

The effects of intra-VTA infusions of exendin-4 on cocaine priming-induced reinstatement of drug-seeking behavior were studied in separate cohorts of rats. Exendin-4 (0.005 and 0.05 µg/100 nl) and vehicle were microinjected bilaterally into the VTA 10 min prior to a priming injection of cocaine (10 mg/kg, i.p.). Using a within-subjects design, each rat served as its own control. To control for potential rank order effects of drug and vehicle administrations, all treatments were counterbalanced across reinstatement test sessions. The doses and time course of administration for each of the aforementioned pharmacological compounds were based on our previous in vivo studies characterizing the effects GLP-1 receptor agonists on cocaine self-administration and food intake in rats (exendin-4; [17, 22] exendin-(9–39); [17, 22] fluoro-Ex-4 [19, 21]).

### Statistics

For all cocaine and sucrose reinstatement experiments, the total mean active and inactive lever responses were analyzed with two-way analysis of variances (ANOVAs). Analyses of body weight and feeding behaviors for the fluoro-Ex-4 dose–response experiment were conducted using repeated measures one-way ANOVAs. Pairwise analyses were made using Bonferroni post hoc tests (*p* < 0.05). Changes in fold mRNA expression of VTA GLP-1 receptors and NTS PPG were analyzed using unpaired *t*-tests.

## Results

### Systemic administration of a GLP-1 receptor agonist dose dependently attenuated cocaine seeking in rats

**Fig. 1:**
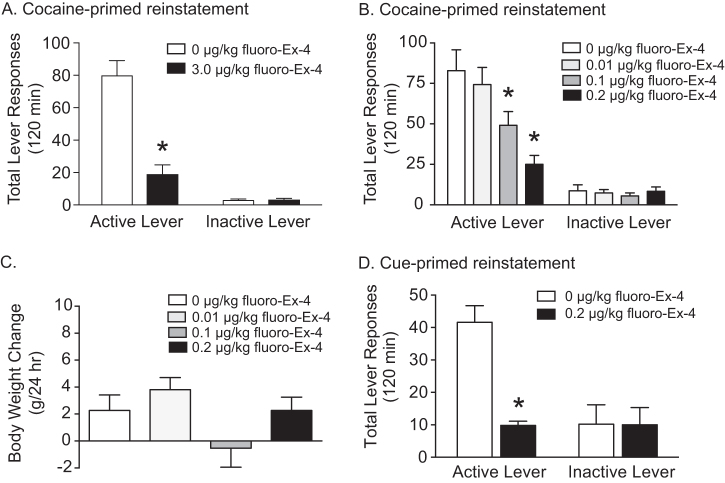
Systemic administration of fluoro-exendin-4 dose dependently attenuated cocaine priming-induced reinstatement of drug-seeking behavior. **a** Cocaine seeking was significantly attenuated in rats pretreated with 3.0 µg/kg fluoro-Ex-4 compared to vehicle-treated controls (*n* = 10/treatment). Since 3.0 µg/kg exendin-4 produces malaise-like effects in rats [20, 21], lower doses of fluoro-Ex-4 were tested to identify doses that selectively reduce cocaine seeking. **b** Doses of fluoro-Ex-4 (0.1 and 0.2 µg/kg) subthreshold for producing malaise-like effects reduced cocaine seeking (*n* = 16/treatment). **c** No effects of systemic fluoro-Ex-4 on 24 h body weight were found in rats pretreated with 0.01, 0.1 and 0.2 µg/kg fluoro-Ex-4 prior to a cocaine priming-induced reinstatement test session (*n* = 16/treatment). **d** Cue-induced reinstatement of cocaine-seeking behavior was significantly attenuated in rats pretreated with 0.2 µg/kg fluoro-Ex-4 compared to vehicle-treated controls (*n* = 5/treatment). *p < 0.05, Bonferroni

Total active and inactive lever responses (mean ± SEM) in rats pretreated with systemic fluoro-Ex-4 (vehicle or 3.0 µg/kg, i.p.; *n* = 10/treatment) prior to a cocaine priming-induced reinstatement test session are shown in Fig. 1a. These data were analyzed with a two-way ANOVA, which revealed significant main effects of treatment (*F*(1,36) = 28.63, *p* < 0.0001) and lever (*F*(1,36) = 66.3, *p* < 0.0001) as well as a significant interaction between lever and treatment (*F*(1,36) = 29.01, *p* < 0.0001). Subsequent pairwise analyses indicated that active lever responses were significantly different between vehicle and 3.0 µg/kg fluoro-Ex-4 (Bonferroni, *p* < 0.05). No significant differences were found on inactive lever responding between treatments.

While peripheral administration of 3.0 µg/kg fluoro-Ex-4 significantly attenuated cocaine seeking, one could argue that the effects of fluoro-Ex-4 on drug seeking were due to malaise-like effects as previous studies have shown that an acute injection of 3.0 µg/kg exendin-4 produces a mild pica response and reduces food intake and body weight in rats [20, 21]. Therefore, we aimed to identify doses of systemic fluoro-Ex-4 that attenuated cocaine seeking and did not produce pica [21] or reduce body weight. We assessed the ability of lower doses of fluoro-Ex-4 (0, 0.01, 0.1, and 0.2 µg/kg, i.p.; *n* = 16/treatment) to attenuate cocaine priming-induced reinstatement of drug seeking behavior. Total active and inactive lever responses (mean ± SEM) from the reinstatement test sessions are shown in Fig. 1b. These data were analyzed with a two-way ANOVA, which revealed significant main effects of treatment (*F*(3,120) = 6.798, *p* < 0.001) and lever [*F*(1,120) = 98.85, *p* < 0.0001] as well as a significant lever×treatment interaction (*F*(3,120) = 6.585, *p* < 0.001). Subsequent pairwise analyses indicated significant decreases between vehicle and 0.1 and 0.2 µg/kg fluoro-Ex-4 on active lever responses (Bonferroni, *p* < 0.05). No significant differences were found on inactive lever responding between treatments. Body weight was measured 24 h after each treatment to determine if peripheral fluoro-Ex-4 at these doses resulted in a reduction of body weight. Mean body weight changes for each treatment are shown in Fig. 1c. There were no significant effects of fluoro-Ex-4 treatment on 24 h body weight in cocaine-experienced rats.

We also investigated the ability of low dose fluoro-Ex-4 to attenuate cue-induced reinstatement of cocaine seeking. Consistent with the effects on cocaine priming-induced reinstatement (Fig. 1b), 0.2 µg/kg fluoro-Ex-4 pretreatment significantly reduced reinstatement of drug-seeking behavior elicited by re-exposure to conditioned cues previously associated with cocaine self-administration (Fig. 1d).

### Doses of systemic fluoro-exendin-4 that attenuated cocaine seeking had no effect on chow intake, number of meals or meal size in cocaine-experienced rats

**Fig. 2:**
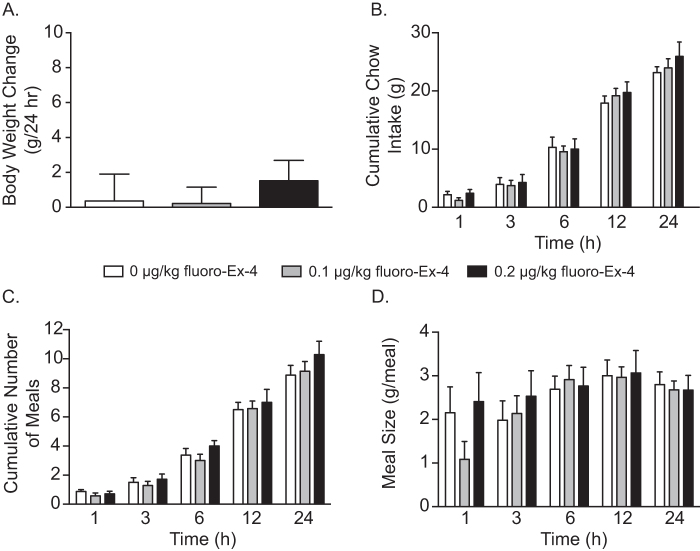
Systemic administration of fluoro-exendin-4 did not affect ad libitum feeding behavior in cocaine-experienced rats. There were no effects of 0.1 and 0.2 µg/kg fluoro-Ex-4 pretreatment on 24 h body weight (**a**) in cocaine-experienced rats. Moreover, fluoro-Ex-4 did not affect cumulative chow intake (**b**), meal frequency (**c**) or meal size (**d**) at any time point following cocaine priming-induced reinstatement test sessions (*n* = 7/treatment)

To further evaluate potential adverse effects of systemic fluoro-Ex-4 on feeding behaviors in cocaine-experienced rats, meal pattern analyses were performed on each reinstatement test day. Meal pattern analyses began immediately after reinstatement test sessions (i.e., 3 h post fluoro-Ex-4 injection), a time point that coincides with decreased food intake in rats treated with higher doses of exendin-4 [21]. Peripheral injections of fluoro-Ex-4 (0.1 and 0.2 µg/kg, i.p.) attenuated cocaine seeking in separate cohorts of rats (*n* = 7/treatment; data not shown but consistent with Fig. 1b) and had no effect on 24 h body weight gain (Fig. 2a) similar to Fig. 1c. Detailed analyses of feeding patterns revealed no effect of fluoro-Ex-4 treatment on cumulative chow intake (Fig. 2b), number of meals (Fig. 2c) and meal size (Fig. 2d) at any time point. Taken together, these data demonstrated that fluoro-Ex-4 attenuated cocaine seeking at doses (0.1 and 0.2 µg/kg) subthreshold for effects on ad libitum chow intake.

### Peripherally administered fluoro-exendin-4 penetrated the brain and was visualized in the VTA

**Fig. 3:**
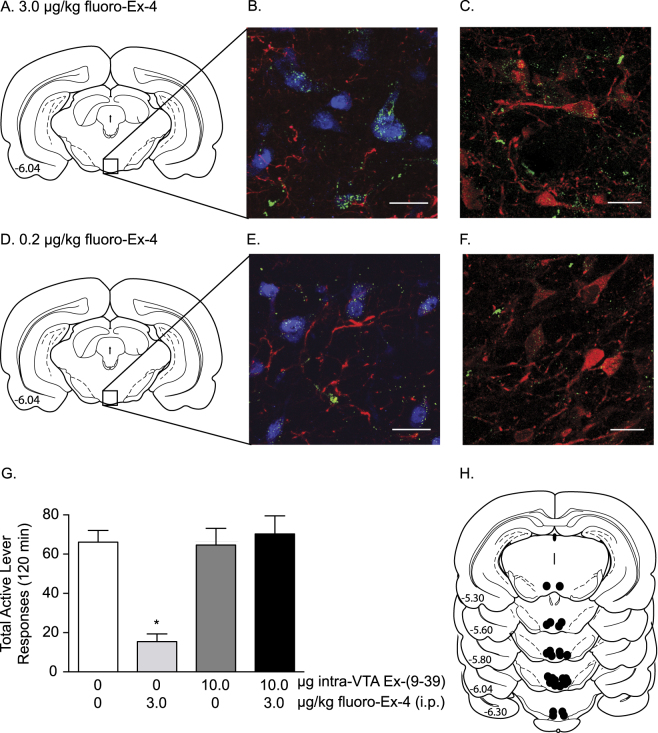
The suppressive effects of systemic fluoro-exendin-4 on cocaine seeking were blocked by antagonism of VTA GLP-1 receptors. Rats pretreated with fluoro-Ex-4 (3.0 µg/kg, *n* = 10) prior to a priming injection of cocaine in Fig. 1a were perfused immediately after the reinstatement test session (3 h post fluoro-Ex-4 infusion). Coronal sections at the level of the midbrain **a** were used to determine whether systemic fluoro-Ex-4 penetrates the brain and localizes in the VTA. **b** 3.0 µg/kg fluoro-Ex-4 (green fluorescence) co-localized with neurons labeled with NeuN (blue fluorescence) and astrocytes labeled with GFAP (red fluorescence) in the VTA. **c** 3.0 µg/kg fluoro-Ex-4 co-localized with tyrosine hydroxylase-labeled dopamine neurons (red fluorescence) in the VTA. The VTA **d** of separate rats treated with 0.2 µg/kg fluoro-Ex-4 were used to determine if lower doses of the GLP-1 receptor agonist that selectively attenuated cocaine seeking also penetrated the brain and localized in the VTA **e** & **f**. Images are compressed z-stacks with a 0.5 µm step size (scale bar: 20 µm). All images shown at 63 × magnification. **g** Intra-VTA infusions of the GLP-1 receptor antagonist exendin-(9–39) (10 µg) prior to a systemic injection of 3.0 µg/kg fluoro-Ex-4 blocked fluoro-Ex-4-mediated reductions in total active lever responses during cocaine reinstatement test sessions (*n* = 13/treatment). **h** Representative coronal sections at the level of the midbrain depict microinjection sites in the VTA. *p < 0.05, Bonferroni

Immediately following the reinstatement test session, rats pretreated with a systemic injection of 3.0 µg/kg fluoro-Ex-4 (Fig. 1a) or 0.2 µg/kg fluoro-Ex-4 (Fig. 1b) were euthanized and their brains removed to determine whether fluoro-Ex-4 was present in the VTA (Fig. 3a, d). Coronal sections of the VTA were immunohistochemically processed to label neurons and astroctyes. Confocal microscopy revealed co-localization of fluoro-Ex-4 with both GFAP-positive astrocytes and NeuN-positive neurons in the VTA (Fig. 3b, e). Separate immunohistochemical analyses to evaluate if fluoro-Ex-4 bound putative GLP-1 receptors expressed on dopamine neurons in the VTA revealed that fluoro-Ex-4 co-localized with tyrosine hydroxylase-positive neurons (Fig. 3c, f).

### Intra-VTA administration of the GLP-1 receptor antagonist exendin-(9–39) prevented the ability of peripheral fluoro-exendin-4 to attenuate cocaine seeking in rats

Our immunohistochemistry findings suggested that the effects of peripheral fluoro-Ex-4 on cocaine seeking are mediated, in part, by activation of GLP-1 receptors in the VTA. To test this hypothesis, the GLP-1 receptor antagonist exendin-(9–39) (Ex-9; 10 µg/100 nl) was administered directly into the VTA prior to a systemic injection of fluoro-Ex-4 (3.0 µg/kg, i.p.) and a cocaine priming-induced reinstatement test session. Using a within-subjects design, there were 4 treatment conditions (intra-VTA vehicle/peripheral vehicle, intra-VTA vehicle/peripheral fluoro-Ex-4, intra-VTA Ex-9/peripheral vehicle, intra-VTA Ex-9/peripheral fluoro-Ex-4; *n* = 13/treatment). Total active lever responses (mean ± SEM) are shown in Fig. 3g. These data were analyzed with a two-way ANOVA which revealed significant main effects of systemic treatment [*F*(1,12) = 18.06, *p* < 0.01] and intra-VTA treatment [*F*(1,12) = 10.94, *p* < 0.01] as well as a significant systemic treatment x intra-VTA treatment interaction [*F*(1,12) = 14.52, *p* < 0.01]. Subsequent pairwise analyses indicated a significant difference in total active lever responding between intra-VTA vehicle/peripheral vehicle, intra-VTA Ex-9/peripheral vehicle, and intra-VTA Ex-9/peripheral fluoro-Ex-4 vs. intra-VTA vehicle/peripheral fluoro-Ex-4-treated rats (Bonferroni, *p* < 0.05). There was no effect of treatment on total inactive lever responding (data not shown). Corresponding microinjection sites in the VTA are shown in Fig. 3h. Taken together, these results suggested that the effects of peripheral fluoro-Ex-4 on the reinstatement of cocaine-seeking behavior were due, in part, to activation of GLP-1 receptors in the VTA.

### Extinction following cocaine self-administration was associated with decreased PPG mRNA expression in the NTS

**Fig. 4:**
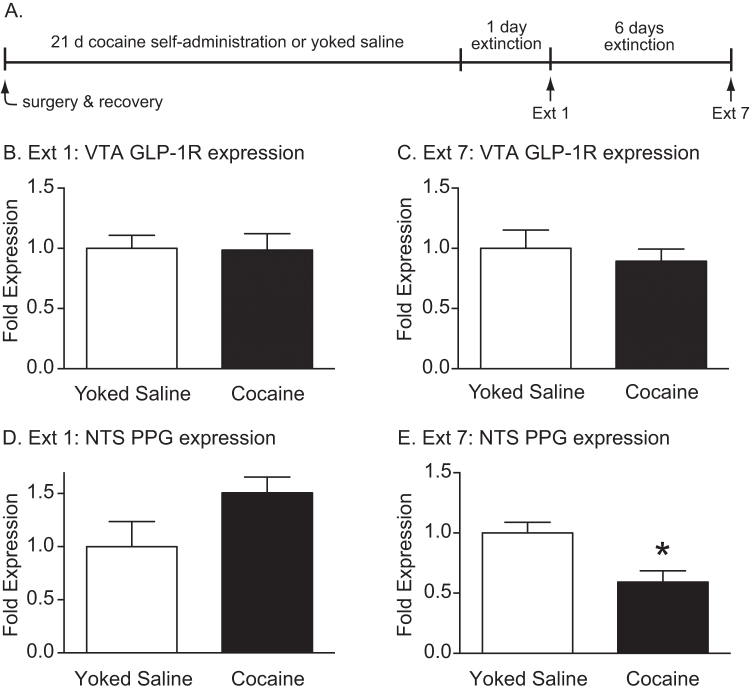
Cocaine self-administration and subsequent extinction reduced endogenous PPG mRNA expression in the NTS. **a** Expression of GLP-1 receptor mRNA transcripts in the VTA and PPG mRNA transcripts in the NTS was quantified following one (Ext1) and seven (Ext7) days of extinction following cocaine self-administration. No differences in GLP-1 receptor mRNA expression in the VTA were found after 1 day of extinction (*n* = 8) **b** or 7 days of extinction (*n* = 7) **c** in cocaine-experienced rats compared to yoked saline controls. **d** While not statistically significant (*p* < 0.09), there was a trend towards increased PPG mRNA expression in the NTS following 1 day of extinction (*n* = 7) in cocaine-experienced rats compared to control rats. **e** In contrast, statistically significant decreases in NTS PPG mRNA expression were found following 7 days of extinction in cocaine-experienced rats compared to yoked saline controls. *p < 0.01, unpaired *t*-tests

Using quantitative real-time PCR, we assessed the effects of cocaine self-administration and subsequent extinction sessions on expression of GLP-1 receptor mRNA transcripts in the VTA and PPG mRNA transcripts in the NTS (Fig. 4a). GLP-1 receptor mRNA expression in the VTA was not changed in cocaine-experienced rats following one (Ext1) and seven (Ext7) days of extinction when compared to yoked saline controls (Fig. 4b, c). While PPG mRNA expression in the NTS was not altered in cocaine-experienced rats following 1 day of extinction (Fig. 4d), NTS PPG mRNA expression was significantly decreased following 7 days of extinction [t(14) = 3.18, *p* < 0.01; *n* = 8/treatment] (Fig. 4e). These results indicated that extinction following cocaine self-administration is associated with decreased endogenous PPG mRNA expression in the NTS.

### Administration of exendin-4 directly into the VTA dose dependently attenuated cocaine seeking in rats

**Fig. 5:**
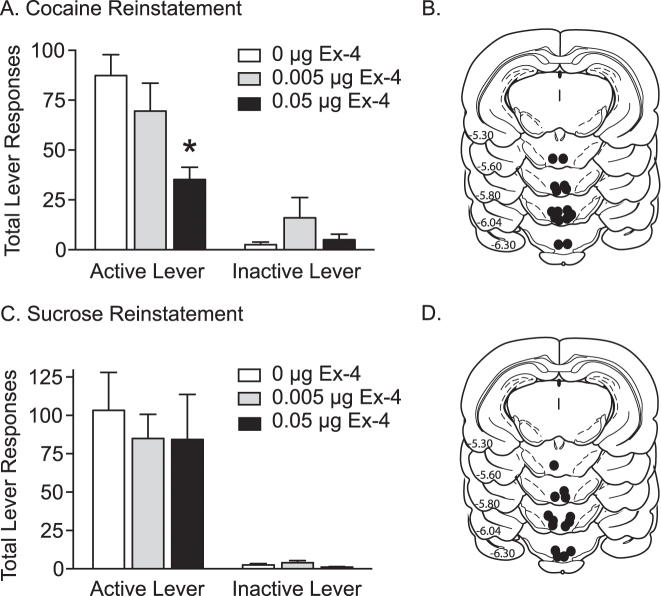
Administration of exendin-4 directly into the VTA dose dependently attenuated cocaine priming-induced reinstatement of drug-seeking behavior. **a** Infusions of exendin-4 (0.05 µg) directly into the VTA prior to a cocaine priming injection reduced active lever responses during subsequent reinstatement test sessions (*n* = 8). The asterisk indicates a significant difference in responding between rats pretreated with 0.05 µg exendin-4 and vehicle (*p < 0.05, Bonferroni). Intra-VTA microinjection sites for the cocaine reinstatement study are shown in **b**. **c** Exendin-4 administration into the VTA had no effect on sucrose seeking (*n* = 7). Microinjection sites corresponding to intra-VTA infusions in sucrose reinstatement test sessions are shown in **d.**

To determine whether increased activation of VTA GLP-1 receptors during extinction is sufficient to attenuate cocaine seeking, exendin-4 was infused directly into the VTA 10 min prior to a cocaine priming-induced reinstatement test session. Total active and inactive lever responses (mean ± SEM) in animals pretreated with exendin-4 (vehicle, 0.005 or 0.05 µg/100 nl, *n* = 8/treatment) are shown in Fig. 5a. These data were analyzed using a two-way ANOVA, which revealed significant main effects of treatment (*F*(2,56) = 6.735, *p* < 0.01) and lever (*F*(1,56) = 75.37, *p* < 0.001) as well as a significant treatment×lever interaction (*F*(2,56) = 6.828, *p* < 0.01). Subsequent pairwise analyses indicated that active lever responses were significantly different between vehicle and 0.05 µg exendin-4 (Bonferroni, *p* < 0.05). No significant differences were found on inactive lever responding between treatments. Microinjection sites corresponding to these experiments are shown in Fig. 5b.

While intra-VTA administration of exendin-4 had no significant effect on inactive lever responding, one could argue that responses were too low to assess the potential rate-suppressant effects of intra-VTA exendin-4 treatment. Total active and inactive lever responses (mean ± SEM) are shown in Fig. 5c for rats pretreated with intra-VTA exendin-4 prior to a sucrose reinstatement test session. There were no effects of intra-VTA exendin-4 on sucrose seeking. Infusion sites within the VTA for these experiments are depicted in Fig. 5d. Taken together with the cocaine reinstatement studies, these data indicated that activation of VTA GLP-1 receptors reduced cocaine seeking and that these effects were not due to deficits in operant responding.

## Discussion

The present study identified a critical role for GLP-1 receptors in cocaine-seeking behavior. Specifically, we showed that systemically administered exendin-4 attenuated the reinstatement of drug-seeking behavior elicited by both cocaine and conditioned cues. Importantly, we identified doses of exendin-4 that selectively reduced cocaine seeking and did not affect food intake or body weight. Moreover, these behaviorally relevant doses of exendin-4 do not produce nausea/malaise-like adverse effects in rodents [21] further highlighting the selectivity of this behavioral response at these doses. We also showed that peripheral exendin-4 was able to cross the blood brain barrier and putatively bind receptors expressed on neurons and astrocytes in the VTA. Indeed, the suppressive effects of peripheral exendin-4 on cocaine seeking were due, in part, to activation of central GLP-1 receptors in the VTA. Moreover, cocaine self-administration and subsequent extinction may reduce endogenous GLP-1 expression in the brain, as evidenced by a significant decrease in PPG mRNA expression in the NTS following 7 days of extinction, a time point that coincides with drug-seeking behavior [23, 24]. Since the NTS sends direct monosynaptic GLP-1-expressing efferent projections to the VTA [22], these results suggest that decreased endogenous GLP-1 release in midbrain areas including the VTA may facilitate cocaine seeking. This hypothesis is supported by our data indicating that infusions of exendin-4 directly into the VTA were sufficient to attenuate the reinstatement of cocaine-seeking behavior. Collectively, these findings identify a novel neural mechanism focused on central GLP-1 receptors that could be targeted to prevent cocaine craving-induced relapse.

### GLP-1 receptor agonists and cocaine addiction

A growing literature indicates that GLP-1 receptors play an important role in addiction-like behaviors and that systemic administration of GLP-1 receptor agonists reduces the rewarding and reinforcing effects of drugs of abuse [9, 11]). For example, peripheral administration of the GLP-1 receptor agonist exendin-4 has been shown to attenuate cocaine-induced conditioned place preference (CPP) and cocaine self-administration in mice [25–27]. Our findings expand on these studies and identified an important role for GLP-1 receptors in an animal model of cocaine craving-induced relapse.

The present study also identified doses of peripheral exendin-4 that selectively attenuated addiction-like behaviors and did not produce adverse effects commonly associated with higher doses in rodents. Indeed, a significant limitation to interpreting results from previous studies of peripheral exendin-4 in preclinical models of drug addiction is the exceedingly high doses of exendin-4 tested. While previous studies have laid the foundation for our understanding of the role of GLP-1 in drug addiction, the doses of peripheral exendin-4 used to pretreat mice in these studies ranged from 3.0 to 100.0 µg/kg [25–27]. Doses of exendin-4 as low as 0.25 µg/kg have been shown to produce malaise-like adverse effects in rats, which can confound subsequent behavioral responses [20, 21]. Moreover, nausea and malaise are common adverse effects associated with high doses of peripherally administered GLP-1 receptor agonists in humans [28]. Since the doses of exendin-4 shown to reduce cocaine CPP and self-administration are likely producing malaise-like effects in mice, it is impossible to draw firm conclusions about the role of GLP-1 receptors in addiction-like behaviors from these previous studies. In contrast, we identified systemic doses of exendin-4 as low as 0.1 and 0.2 µg/kg that were sufficient to attenuate cocaine seeking in rats. Importantly, these doses were subthreshold for effects on feeding behavior in cocaine-experienced rats (present findings) and do not produce malaise-like effects in rats [20, 21]. The translational implications of these findings are profound in that they support potential therapeutic approaches toward the specific use of GLP-1 receptor agonists for the treatment of cocaine craving and relapse.

In addition to causing nausea and malaise-like effects, peripheral administration of exendin-4 at doses as low as 0.25 µg/kg have been shown to significantly decrease food intake and body weight in rats [20, 21]. These findings further confound prior studies that examined the effects of exendin-4 on drug-mediated behaviors because they indicate that doses of exendin-4 greater than 0.25 µg/kg reduce motivated behaviors generally and not drug reinforcement specifically. Furthermore, reductions in ad libitum food intake and body weight limit the therapeutic potential of GLP-1 receptor agonists for use in human addicts. Our novel findings clearly identified doses of exendin-4 (0.1 and 0.2 µg/kg) that selectively attenuated cocaine seeking and did not affect ad libitum food intake, meal patterns or body weight in cocaine-experienced rats. These data support selective effects of a peripherally administered GLP-1 receptor agonist on drug reinforcement. Consistent with our peripheral exendin-4 dose–response study, we also showed that exendin-4 reduced cocaine seeking when infused directly into the VTA at a dose (0.05 µg) that does not affect ad libitum chow intake or promote malaise-like effects in rats [22, 29, 30]. Moreover, intra-VTA infusion of exendin-4 did not alter sucrose seeking further supporting the selectivity of lower doses to reduce cocaine seeking. There is some evidence that administration of 0.05 µg exendin-4 into the VTA reduces operant responding for palatable food [30]. However, these effects are transient and do not persist with more prolonged operant sessions [17, 30]. Thus, the present study showed that a GLP-1 receptor agonist reduced cocaine seeking at doses not associated with common adverse effects in rodents.

### Central GLP-1 receptors and cocaine seeking

Mutant mice lacking GLP-1 receptors have enhanced cocaine CPP compared to wild-type controls [31]. These results suggest that activation of central GLP-1 receptors may function to reduce the rewarding effects of cocaine. Consistent with this hypothesis, we recently showed that direct activation of GLP-1 receptors in the VTA is sufficient to reduce cocaine self-administration in rats [17]. We expanded upon these studies here and showed that the intake suppressive effects of peripheral exendin-4 on cocaine seeking were due to activation of GLP-1 receptors in the VTA and that direct activation of VTA GLP-1 receptors was sufficient to reduce cocaine seeking.

The current data clearly support a role for VTA GLP-1 receptors in cocaine seeking, but it is possible and in fact likely that GLP-1 receptors expressed in other brain regions also play an important role in cocaine seeking. GLP-1 receptors are expressed ubiquitously throughout the rodent brain [15] including nuclei known to regulate drug-seeking behavior. Viral-mediated re-expression of GLP-1 receptors in the lateral septum of constitutive GLP-1 receptor knockout mice attenuates cocaine CPP [31] indicating that the rewarding effects of cocaine are mediated, in part, by enhanced GLP-1 signaling in this nucleus. GLP-1 receptors are also expressed in the nucleus accumbens, hippocampus, habenula and amygdala [15, 32] and future studies are required to define the exact role of GLP-1 signaling in these nuclei in addiction-like behaviors.

The exact mechanism(s) by which GLP-1 receptor activation in the VTA attenuates cocaine seeking are unknown. Doses of peripheral exendin-4 that reduce cocaine CPP and self-administration attenuate cocaine-mediated increases in extracellular dopamine in the nucleus accumbens [26, 27]. Since increased dopamine signaling in the accumbens promotes cocaine seeking [16, 33, 34], these results suggest that reduced cocaine seeking following peripheral exendin-4 administration may involve decreased extracellular dopamine levels in the nucleus accumbens. The present findings also indicated that peripheral exendin-4 bound putative GLP-1 receptors expressed on neurons and astrocytes in the VTA. However, it is not clear how exactly GLP-1 receptor activation in the VTA may reduce dopamine cell firing. GLP-1 receptors are predominantly Gs-coupled receptors (although they can activate Gq and Gi proteins as well) that are expressed on both pre- and post-synaptic sites in the VTA [4, 35]. While the exact phenotypes of GLP-1 receptor-expressing cells in the VTA must be further characterized, there is some evidence that GLP-1 receptor activation increases glutamate release in the VTA of drug-naïve rats [29]. GLP-1 receptor activation also enhances GABAA receptor-mediated currents in the drug-naïve brain [36–38], suggesting a possible GABA-mediated mechanism in the VTA that may underlie the suppressive effects of exendin-4 on cocaine seeking. Furthermore, a recent study showed that GLP-1 receptor agonists increase activation of astrocytes in the hindbrain [19]. It is intriguing to think that increased activation of GLP-1 receptors expressed on astrocytes in the VTA may regulate glutamate homeostasis and drug-seeking behavior. The effects of GLP-1 receptor activation on dopamine cell firing in the VTA are likely to be complex and may differ between drug-naïve and cocaine-experienced brains. Therefore, future studies are required to determine the cellular and neurophysiological mechanisms through which activation of GLP-1 receptors in the VTA suppresses cocaine-seeking behavior.

### Extinction following cocaine self-administration dynamically regulated endogenous PPG mRNA expression in the NTS

To date no studies have examined the effects of cocaine self-administration and subsequent extinction on endogenous central GLP-1 signaling. We have previously reported that cocaine activates GLP-1-expressing neurons in the NTS and that increased VTA GLP-1 signaling in the brain may function as a homeostatic response to reduce cocaine self-administration [17]. To expand upon these data, we examined the effects of 1 day and 7 days of extinction following 21 days of cocaine self-administration on GLP-1 receptor mRNA expression in the VTA. We also quantified expression of PPG mRNA in the NTS. PPG is the protein precursor to GLP-1 in NTS neurons [4]. Using quantitative real-time PCR, we found no effects of extinction on VTA GLP-1 receptor mRNA expression. In contrast, we found a non-significant trend (*P* = 0.09) toward increased PPG mRNA expression in the NTS during acute withdrawal from cocaine self-administration. These results are consistent with our previous study showing that cocaine increases activation of PPG-expressing neurons in the NTS [17] and together suggest that increased endogenous PPG mRNA expression in the NTS may represent a homeostatic compensatory response to cocaine self-administration that serves to reduce further drug taking. Interestingly, we observed a significant decrease in NTS PPG mRNA expression following 7 days of extinction, a time point that coincides with maximal drug-seeking behavior [23, 24]. Since activation of central GLP-1 receptors may serve as a ‘brake’ on cocaine self-administration, this decrease in endogenous PPG mRNA expression in the caudal brain may facilitate drug seeking during extinction. Support for this hypothesis comes from the present study showing that direct activation of VTA GLP-1 receptors was sufficient to reduce cocaine priming-induced reinstatement of drug-seeking behavior. Our data are consistent with previous literature demonstrating that reduced PPG mRNA expression in the NTS increases the rewarding value of food. Viral-mediated knockdown of PPG mRNA expression in the NTS produces hyperphagia and exacerbates high fat diet-induced obesity [39]. Due to shared mechanisms mediating the rewarding properties of food and drugs of abuse [40, 41], our data further support a role for central GLP-1 signaling in regulating motivated behaviors.

## Conclusion

The present study identified a novel role for central GLP-1 receptors in an animal model of relapse. Moreover, we have identified doses of the GLP-1 receptor agonist exendin-4 that selectively reduced cocaine seeking and did not produce adverse effects in rats. Since GLP-1 receptor agonists are FDA-approved for treating diabetes type II and obesity, these findings suggest that exendin-4 could be re-purposed as an anti-relapse medication. In addition, we provided evidence that extinction following cocaine self-administration decreased endogenous PPG mRNA expression in the brain, which may facilitate drug-seeking behavior.

## Electronic supplementary material

Supplemental Materials
