# Supplementary material for: Glucagon-like peptide-1 receptor activation in the ventral tegmental area attenuates cocaine seeking in rats
Source: Neuropsychopharmacology. 2018 Feb 14;43(10):2000–8. doi: 10.1038/s41386-018-0010-3 (PMC6098066; doi:10.1038/s41386-018-0010-3)
Supplement: Supplementary file 1 — Supplemental Materials [file 41386_2018_10_MOESM1_ESM.docx]

**Supplemental Materials and Methods**

*Drugs*

Cocaine was obtained from the National Institute on Drug Abuse (Rockville, MD) and dissolved in bacteriostatic 0.9% saline. Exendin-4 and exendin-(9-39) were purchased from the American Peptide Company (Sunnyvale, CA) and were dissolved in artificial cerebrospinal fluid (aCSF; Harvard Apparatus, Holliston, MA). Fluoro-Ex-4 was purchased from AnaSpec (Fremont, CA) and dissolved in bacteriostatic 0.9% saline. The doses and time course of administration for each of the aforementioned pharmacological compounds were based on the following systemic and intra-cranial microinjection experiments in rats: exendin-4 (Alhadeff *et al*, 2012; Schmidt *et al*, 2016), exendin-(9-39) (Alhadeff *et al*, 2012; Schmidt *et al*, 2016) and fluoro-Ex-4 (Kanoski *et al*, 2012; Reiner *et al*, 2016).

*Surgery*

Rats were handled daily and allowed one week to acclimate to their home cages upon arrival. Prior to surgery, rats were anesthetized with 80 mg/kg ketamine (Midwest Veterinary Supply, Valley Forge, PA) and 12 mg/kg xylazine (Sigma-Aldrich/RBI, St. Louis, MO). An indwelling catheter (SAI Infusion Technologies, Lake Villa, IL) was inserted into the right jugular vein and sutured in place. The catheter was routed to a mesh backmount that was implanted subcutaneously above the shoulder blades. To prevent infection and maintain patency, catheters were flushed daily with 0.2 ml of the antibiotic Timentin (0.93 mg/ml; Fisher, Pittsburgh, PA) dissolved in heparinized 0.9% saline (Butler Schein, Dublin, OH). When not in use, catheters were sealed with plastic obturators.

After catheter insertion, some rats were then immediately mounted in a stereotaxic apparatus (Kopf Instruments, CA) and implanted with cannulae for intra-cranial microinjections. Bilateral guide cannulae (26 gauge; 16 mm; Plastics One, Roanoke, VA) were implanted 2mm dorsal to the VTA and cemented in place by affixing dental acrylic to stainless steel screws secured in the skull. The coordinates for the ventral ends of the guide cannulae, relative to bregma according to the atlas of Paxinos and Watson (1997), were as follows: -5.8 mm A/P, ±0.5 mm M/L, and -6.6 mm D/V. An obturator (33 gauge; Plastics One) was inserted into each guide cannula to prevent occlusion.

*Cocaine self-administration, extinction and reinstatement of cocaine seeking*

Rats were allowed 7 days to recover from surgery before behavioral testing commenced. Initially, rats were placed in operant conditioning chambers and allowed to lever-press for intravenous infusions of cocaine (0.25 mg/kg/infusion, infused over a 5 s period) on a fixed-ratio 1 (FR1) schedule of reinforcement. Rats were allowed to self-administer a maximum of 30 injections per 120 min operant session. Once a rat achieved at least 20 infusions of cocaine in a single daily operant session under the FR1 schedule, the subject was switched to a fixed-ratio 5 (FR5) schedule of reinforcement. The maximum number of injections was again limited to 30 per daily self-administration session under the FR5 schedule. For both FR1 and FR5 schedules, a 20 s time-out period followed each cocaine infusion, during which time active lever responses were tabulated but had no scheduled consequences. Responses made on the inactive lever, which had no scheduled consequences, were also recorded during both the FR1 and FR5 training sessions. Following 21 days of daily cocaine self-administration sessions, drug-taking behavior was extinguished by replacing the cocaine solution with 0.9% saline. Daily extinction sessions continued until responding on the active lever was <15% of the total active lever responses completed on the last day of cocaine self-administration. Typically, it took ~7 days for rats to meet this criterion. Once cocaine self-administration was extinguished, rats entered the reinstatement phase of the experiment. During reinstatement test sessions, satisfaction of the response requirement (i.e., five presses on the active lever) resulted in an infusion of saline rather than cocaine. Using a between-sessions reinstatement procedure, each reinstatement test session was followed by extinction sessions until responding was again <15% of the total active lever responses completed on the last day of cocaine self-administration. Generally, 1–2 days of extinction were necessary to reach extinction criterion between reinstatement test sessions.

Cue-induced reinstatement of cocaine-seeking behavior was tested in a separate cohort of rats. The same self-administration procedure was used as described above with the addition of contingent light cues. Each cocaine infusion was associated with concurrent illumination of a cue light located directly above the active lever for 5 s. Following 21 days of cocaine self-administration sessions, drug-taking behavior was extinguished by replacing the cocaine solution with saline and turning off the drug-paired cue light. Once cocaine self-administration was extinguished (operationally defined as <15% of the total active lever responses completed on the last day of cocaine self-administration), rats entered the reinstatement phase of the experiment and the ability of the cue light to reinstate drug-seeking behavior was assessed. During reinstatement test sessions, every 5^th^ lever press resulted in an infusion of saline and illumination of the cue light previously paired with cocaine taking during the self-administration phase of the experiment. The effects of systemic vehicle and fluoro-Ex-4 (0.2 µg/kg, i.p.) on cue-induced reinstatement of cocaine-seeking behavior were tested using a counterbalanced, within-subjects design.

*Sucrose self-administration, extinction and reinstatement of sucrose seeking*

Potential nonspecific rate-suppressing effects of intra-VTA exendin-4 were evaluated by assessing the influence of exendin-4 on the reinstatement of sucrose-seeking behavior. Separate cohorts of rats were trained initially to self-administer 45 mg sucrose pellets (Research Diets, New Brunswick, NJ) on a FR1 schedule of reinforcement during daily one hour operant sessions. Once rats achieved stable responding for sucrose (defined as <20% variation in responding over 3 consecutive days) on the FR1 schedule of reinforcement, the response requirement was increased to an FR5 schedule of reinforcement. Rats were limited to 30 sucrose pellets within each daily operant session and were restricted to ~20-25 g of lab chow (Harlan Teklad, Wilmington, DE) daily in their home cages for the duration of the experiment. Water was available *ad libitum* in the home cage.

After two weeks of sucrose-maintained responding on an FR5 schedule of reinforcement, rats underwent an extinction phase where active lever pressing no longer resulted in sucrose delivery. Once active lever responding decreased to <15% of the maximum number of responses completed on the last day of sucrose self-administration, rats proceeded to reinstatement testing. Exendin-4 (0.005 and 0.05 µg/100 nl) and vehicle were microinjected into the VTA 10 min prior to the beginning of the reinstatement test sessions. Using a within-subjects design, each animal served as its own control and doses were counterbalanced across test sessions. The experimenter remotely administered one sucrose pellet every two min for the first 10 min of the reinstatement session. A between-session procedure was used so that each daily reinstatement test session was followed by an extinction session the following day until responding was again <15% of the total active lever responses maintained by sucrose.

*Ad libitum food intake*

To assess the effects of fluoro-Ex-4 on chow intake in cocaine-experienced rats, a separate group of rats was housed in a custom-made automated feedometer during the extinction and reinstatement phases of the experiment. Rats were housed individually in hanging wire cages each with a small access hole leading to a food cup resting on an electronic scale as we have described previously (Alhadeff *et al*, 2016; Reiner *et al*, 2016). Each rat was pretreated with vehicle or fluoro-Ex-4 (0.1  or 0.2 μg/kg) one hour prior to a cocaine priming-induced reinstatement test session. Rats were returned to the feedometer immediately following the reinstatement session and given *ad libitum* access to normal chow (Purina LabDiet 5001, Purina, St. Louis, MO). Chow intake and meal patterns were quantified by measuring the weight of each food cup using computer software (LabView) every 10 s for 24 h. Feeding measurements were recorded 1, 3, 6, 12 and 24  h post session (4, 6, 9, 15 and 27 h post infusion). Total body weight and water intake were measured 24 h post session (27 h post infusion). Cumulative food intake and meal patterns were analyzed, with a meal defined as ingestion of at least 0.25 g of food with a minimum of 10 min between feeding bouts (Alhadeff *et al*, 2016; Reiner *et al*, 2016).

*Verification of cannula placements*

After completion of all VTA microinjection experiments, rats were given an overdose of pentobarbital (100 mg/kg, i.p.). Brains were removed and drop fixed in 10% formalin. Coronal sections (100 µm) were taken at the level of the VTA with a vibratome and mounted on gelatin-coated slides. An individual blinded to behavioral responses verified microinjection sites using light microscopy. Rats with cannula placements outside of the VTA and/or excessive mechanical damage were excluded from subsequent data analyses.

*Immunohistochemistry*

Rats pretreated with 0.2 or 3.0 µg/kg fluoro-Ex-4 (i.p.) were deeply anesthetized and transcardially perfused with 0.1 M PBS, pH 7.4, followed with 4% formalin in 0.1 M PBS immediately following their cocaine priming-induced reinstatement test session (i.e., three hours post infusion). Once brains were removed, they were postfixed overnight in 4% formalin in 0.1 M PBS and then cryoprotected in 20% sucrose in 0.1 M PBS at 4^o^C for three days. Coronal sections (30 µm) were then taken at the level of the VTA using a cryostat (Leica 3050S; Leica Corp., Deerfield, IL). Brain sections were stored in 0.1 M PBS at 4^o^C until processed.

Immunohistochemistry was performed on free-floating coronal sections containing the VTA according to modified procedures from previously published studies (Reiner *et al*, 2016; Schmidt *et al*, 2016). Briefly, sections were washed with 1% sodium borohydride followed by 0.1 M PBS. Sections were then blocked in 0.1 M PBS containing 5% normal donkey serum and 0.2% Triton-X for 1 h at room temperature. Sections were incubated in primary antibodies overnight, and then, following a PBS rinse, they were incubated in secondary antibodies for 2 h. The primary antibodies used were rabbit anti-NeuN (1:1000; ab177487, Abcam, Cambridge, UK), goat anti-GFAP (1:1000; ab53554, Abcam, Cambridge, UK) and rabbit anti-tyrosine hydroxylase (1:1000; 2792; Cell Signaling, Danvers, MA). Secondary antibodies were donkey anti-goat Alexa Fluor 594 (1:500), donkey anti-rabbit Alexa Fluor 647 (1:500) and donkey anti-rabbit Alexa Fluor 594 (1:500) from Jackson ImmunoResearch (West Grove, PA). Sections were then washed and mounted onto glass slides and coverslipped using Vectashield (Vector Laboratories; Burlingame, CA). Sections were visualized with a Leica SP5 X confocal microscope using the 20x and 63x oil-immersion objectives along with 488, 594 and 633 nm laser lines. Image z-stacks were captured with a 2–3x optical zoom at the 63x oil-immersion objective with a step size of 0.5 μm.

*Quantitative real-time PCR, cocaine self-administration and yoked saline controls*

Separate rats underwent jugular catheterization as described above. Following a recovery period, the rats were randomly assigned to one of two groups: cocaine-experimental or yoked saline controls. Each rat allowed to respond for contingent cocaine infusions was paired with a yoked rat that received infusions of saline. While lever pressing for the saline-yoked rats had no scheduled consequences, these rats received the same number and temporal pattern of infusions as self-administered by their paired cocaine-experimental rat. Cocaine-experimental rats were allowed to lever press for intravenous cocaine infusions on a FR1 schedule as described above for a total of 21 days.

To assess the effects of cocaine self-administration and extinction on expression of VTA GLP-1 receptors and PPG in the NTS, rats were sacrificed either immediately after the first extinction session (Ext1) or following 7 consecutive days of extinction (Ext7). Brains were collected and flash frozen in -20°C isopentane and stored at -80°C. Brains were subsequently mounted on a cryostat (Leica 3050S; Leica Corp., Deerfield, IL) and coronal sections at the levels of the NTS and VTA were taken. Bilateral 1mm^3^ micropunches of the VTA and NTS were collected for quantitative real-time PCR to determine GLP-1 receptor and PPG expression, respectively. mRNA expression was quantified using Taqman gene expression kits (GLP-1R: Rn00562406_m1; PPG: Rn00562293_m1; GAPDH: Rn01775763_g1; ThermoFisher Scientific, Waltham, MA). qPCR was conducted using an Eppendorf Mastercycler ep realplex2 and the comparative threshold cycle method was used to quantify relative mRNA expression. Relative fold-expression of VTA GLP-1 receptor and NTS PPG transcripts at Ext1 and Ext7 were normalized to yoked saline controls and GAPDH levels.

**References**

Alhadeff AL, Mergler BD, Zimmer DJ, Turner CA, Reiner DJ, Schmidt HD*, et al* (2016). Endogenous Glucagon-like Peptide-1 Receptor Signaling in the Nucleus Tractus Solitarius is Required for Food Intake Control. *Neuropsychopharmacology : official publication of the American College of Neuropsychopharmacology*.

Alhadeff AL, Rupprecht LE, Hayes MR (2012). GLP-1 neurons in the nucleus of the solitary tract project directly to the ventral tegmental area and nucleus accumbens to control for food intake. *Endocrinology* **153**(2): 647-658.

Kanoski SE, Rupprecht LE, Fortin SM, De Jonghe BC, Hayes MR (2012). The role of nausea in food intake and body weight suppression by peripheral GLP-1 receptor agonists, exendin-4 and liraglutide. *Neuropharmacology* **62**(5-6): 1916-1927.

Paxinos G, Watson C (1997). *The rat brain in stereotaxic coordinates* Academic Press: New York.

Reiner DJ, Mietlicki-Baase EG, McGrath LE, Zimmer DJ, Bence KK, Sousa GL*, et al* (2016). Astrocytes Regulate GLP-1 Receptor-Mediated Effects on Energy Balance. *J Neurosci* **36**(12): 3531-3540.

Schmidt HD, Mietlicki-Baase EG, Ige KY, Maurer JJ, Reiner DJ, Zimmer DJ*, et al* (2016). Glucagon-Like Peptide-1 Receptor Activation in the Ventral Tegmental Area Decreases the Reinforcing Efficacy of Cocaine. *Neuropsychopharmacology : official publication of the American College of Neuropsychopharmacology* **41**(7): 1917-1928.
